# Supplementary material for: Assessment of mastitis in camel using high-throughput sequencing
Source: PLoS One. 2022 Dec 7;17(12):e0278456. doi: 10.1371/journal.pone.0278456 (PMC9728900; doi:10.1371/journal.pone.0278456)
Supplement: S1 Appendix — (PDF) [file pone.0278456.s001.pdf]

Supporting Information

Assessment of mastitis in camel using high-throughput sequencing

Rita Rahmeh[1]*, Abrar Akbar[1], Husam Alomirah[1], Mohamed Kishk[1], Abdulaziz Al-Ateeqi[1], Anisha Shajan[1], Thnayan Alonaizi[1], Alfonso Esposito[2]

[1]Environment & Life Sciences Research Center, Kuwait Institute for Scientific Research.

[2] International Centre for Genetic Engineering and Biotechnology, Trieste, Italy

*Corresponding author

E-mail: rrahmeh@kisr.edu.kw (RR)

S1 Table. Summary of camels age, parity, and management

Sample ID	Camel Age	Parity
CM-41	3	11
CM-43	4	14
CM-44	4	14
CM-45	3	11
CM-46	3	11
CM-47	4	14
CM-48	1	5
CM-49	3	11
CM-53	3	11
CM-54	4	14
CM-123	2	8
CM-127	3	11
CM-129	4	14
CM-135	2	8
CM-138	5	16
CM-139	2	8
CM-56M	4	14
CM-59M	5	16
CM-69M	3	11
CM-70M	3	11
CM-125M	1	5
CM-128M	5	16
CM-131M	6	19
CM-142M	6	19
CM-148M	5	16

Management system: The management system for camel in Kuwait is the traditional nomadic system. The camels don’t share food and water with other animals. The milking process of female camels is executed by employers. All camels feed twice daily, in the early morning and the evening. They were also allowed to graze in the desert in the daytime. The ration given to the camels is about 50% forages such as wheat straw and alfalfa and about 50% pulse concentrate feeding (barley, Maze, and wheat Bran).

S2 Table. Tabular summary of the impact of pipeline steps on read pair / ASV counts for healthy and mastitis (M) camel milk samples after sequencing of the V3-V4 region of 16s rRNA gene.

Sample ID	Input read pairs	Recognized read pairs	Filtered read pairs	Merged read pairs	Non-chimeric	With taxonomy
CM-41	114065	109936	109851	51519	50586	50379
CM-43	123103	118781	118698	71321	70940	70911
CM-44	61218	53490	53201	51218	50904	50732
CM-45	59094	51236	50946	47158	46676	46562
CM-46	90638	66955	66896	65933	65598	65257
CM-47	81947	58491	58187	56117	56012	55801
CM-48	59652	42713	42518	40321	39851	39790
CM-49	72241	70228	70155	68828	68777	68381
CM-53	88778	85469	85406	82516	82357	82018
CM-54	102229	98294	98203	93943	83498	83489
CM-123	69499	67087	67020	64625	64577	64350
CM-127	100774	98095	98015	96071	95967	95963
CM-129	104831	101056	100980	94536	93091	92923
CM-135	66242	63779	63721	62511	62379	62379
CM-138	78506	76045	75980	70558	64640	64640
CM-139	120780	116883	116792	113242	112340	112230
CM-56M	102229	67467	67412	65997	65861	65775
CM-59M	96187	56258	55949	46655	46655	46655
CM-69M	95228	85886	85437	70147	70122	70122
CM-70M	108034	104039	103965	78572	78257	78199
CM-125M	150430	145281	145235	112226	112160	112158
CM-128M	152593	147839	147794	82719	80674	80674
CM-131M	50560	45557	45328	42687	42587	42127
CM-142M	93902	91241	91153	88362	85792	85765
CM-148M	110946	68283	67898	62637	60413	60402

S3 Table. Tabular summary of the impact of pipeline steps on read pair / ASV counts for healthy and mastitis (M) camel milk samples after sequencing of the ITS Region.

Sample ID	Input read pairs	Recognized read pairs	Filtered read pairs	Merged read pairs	Non-chimeric	With taxonomy
CM-34	136139	132966	132916	119362	119362	119362
CM-41	124641	121662	121613	105835	105835	104192
CM-43	120303	116812	116752	102799	102799	100981
CM-44	132045	128043	127972	112217	111868	111864
CM-46	155313	151240	151170	128012	128012	128008
CM-47	125660	123611	123557	107181	107178	106724
CM-48	151736	147647	147588	123546	123546	123465
CM-49	141218	137833	137761	101160	101160	100790
CM-53	129728	125671	125609	114055	114055	113721
CM-54	90699	87456	87409	81736	81643	80925
CM-123	170834	167873	167789	145561	145561	145561
CM-127	190034	186503	186425	163836	163836	163685
CM-129	104874	101765	101710	93763	93763	91651
CM-139	118341	114087	114035	103315	103251	103008
CM-56M	127669	124296	124248	117586	117410	117325
CM-125M	124717	122399	122353	110396	110396	110232
CM-128M	129653	126103	126038	118169	118169	117659
CM-148M	33004	32201	32043	31192	31192	31084

S4 Table. Taxon Frequencies Summarized by Class and Genus for Healthy Milk Samples at the genus level

Phylum	Genus	CM-139	CM-138	CM-135	CM-129	CM-127	CM-123	CM-54	CM-53	CM-49	CM-48	CM-47	CM-46	CM-45	CM-44	CM-43	CM-41
Actinobacteriota	*Brachybacterium*	3%	7%	0%	4%	1%	0%	0%	0%	0%	0%	0%	0%	0%	0%	1%	4%
Actinobacteriota	*Corynebacterium*	1%	4%	0%	1%	0%	40%	0%	1%	7%	1%	0%	5%	0%	1%	0%	1%
Actinobacteriota	*Enteractinococcus*	2%	5%	0%	3%	0%	0%	0%	0%	0%	0%	0%	1%	0%	3%	11%	0%
Actinobacteriota	*Glutamicibacter*	23%	43%	1%	24%	2%	4%	1%	2%	0%	0%	0%	0%	1%	2%	5%	5%
Proteobacteria	*Sphingomonas*	0%	0%	1%	0%	1%	0%	0%	0%	10%	5%	6%	5%	0%	4%	1%	1%
Proteobacteria	*uncl. Sphingomonadaceae*	0%	0%	7%	0%	5%	1%	0%	0%	8%	2%	5%	7%	1%	5%	0%	0%
Firmicutes	*Atopostipes*	2%	1%	0%	2%	0%	0%	0%	0%	0%	0%	0%	0%	0%	1%	12%	6%
Firmicutes	*Jeotgalicoccus*	6%	4%	0%	5%	1%	1%	0%	0%	0%	0%	0%	0%	0%	1%	4%	1%
Firmicutes	*Lactobacillus*	0%	0%	0%	0%	0%	2%	0%	0%	13%	10%	10%	10%	8%	5%	3%	21%
Firmicutes	*Lactococcus*	0%	0%	0%	0%	0%	0%	0%	0%	0%	0%	0%	0%	0%	0%	0%	0%
Firmicutes	*Paenibacillus*	1%	1%	18%	1%	22%	3%	0%	0%	8%	1%	7%	5%	0%	5%	1%	0%
Firmicutes	*Pediococcus*	1%	0%	5%	1%	1%	10%	0%	0%	5%	2%	2%	6%	2%	5%	0%	1%
Firmicutes	Staphylococcus	4%	1%	0%	1%	15%	1%	0%	0%	2%	0%	1%	1%	26%	3%	27%	1%
Firmicutes	Streptococcus	2%	0%	1%	1%	0%	0%	0%	1%	3%	27%	0%	1%	0%	1%	11%	2%
Proteobacteria	*Acinetobacter*	1%	0%	0%	2%	0%	0%	32%	63%	0%	2%	1%	1%	0%	0%	1%	1%
Proteobacteria	*Klebsiella*	0%	0%	0%	0%	0%	0%	0%	0%	0%	0%	0%	0%	0%	0%	0%	0%
Proteobacteria	*Moraxella*	2%	0%	5%	1%	0%	0%	0%	10%	3%	5%	0%	6%	0%	4%	0%	5%
Proteobacteria	*Pseudomonas*	1%	0%	2%	1%	0%	2%	62%	0%	2%	5%	12%	4%	0%	3%	0%	0%
Proteobacteria	*Schlegelella*	1%	1%	29%	1%	29%	7%	0%	1%	10%	3%	8%	6%	1%	8%	2%	0%
Proteobacteria	*uncl. Comamonadaceae*	0%	0%	8%	0%	9%	1%	0%	1%	3%	0%	2%	3%	0%	2%	0%	0%
Proteobacteria	*uncl. Enterobacteriaceae*	0%	0%	0%	0%	0%	0%	2%	6%	0%	0%	2%	0%	1%	1%	0%	0%
Others	*Others*	49%	32%	22%	53%	14%	25%	2%	15%	24%	36%	44%	40%	59%	46%	19%	51%

S5 Table. Taxon Frequencies Summarized by Class and Genus for Mastitis Milk Samples at the genus level

Phylum	Genus	CM-148M	CM-142M	CM-131M	CM-128M	CM-125M	CM-70M	CM-69M	CM-59M	CM-56M
Actinobacteriota	*Brachybacterium*	0%	11%	1%	4%	0%	0%	0%	0%	1%
Actinobacteriota	*Corynebacterium*	0%	6%	8%	1%	0%	0%	0%	0%	2%
Actinobacteriota	*Enteractinococcus*	0%	6%	0%	2%	0%	1%	0%	0%	0%
Actinobacteriota	*Glutamicibacter*	0%	4%	7%	45%	3%	0%	1%	2%	8%
Proteobacteria	*Sphingomonas*	0%	0%	0%	0%	1%	0%	0%	0%	0%
Proteobacteria	*uncl. Sphingomonadaceae*	1%	0%	1%	0%	5%	0%	0%	0%	1%
Firmicutes	*Atopostipes*	0%	2%	0%	3%	0%	1%	0%	0%	0%
Firmicutes	*Jeotgalicoccus*	0%	5%	2%	7%	1%	5%	0%	0%	1%
Firmicutes	*Lactobacillus*	0%	0%	0%	0%	0%	0%	0%	0%	1%
Firmicutes	*Lactococcus*	0%	0%	0%	0%	29%	0%	0%	0%	0%
Firmicutes	*Paenibacillus*	14%	0%	1%	0%	19%	0%	0%	0%	0%
Firmicutes	*Pediococcus*	0%	0%	2%	0%	1%	0%	0%	0%	0%
Firmicutes	*Staphylococcus*	0%	3%	4%	2%	0%	81%	0%	0%	0%
Firmicutes	*Streptococcus*	0%	8%	2%	0%	0%	0%	99%	1%	0%
Proteobacteria	*Acinetobacter*	0%	0%	1%	5%	0%	0%	0%	2%	46%
Proteobacteria	*Klebsiella*	0%	0%	0%	0%	0%	0%	0%	26%	0%
Proteobacteria	*Moraxella*	0%	0%	1%	0%	0%	0%	0%	0%	2%
Proteobacteria	*Pseudomonas*	0%	0%	23%	1%	0%	0%	0%	1%	7%
Proteobacteria	*Schlegelella*	78%	0%	2%	0%	26%	0%	0%	0%	0%
Proteobacteria	*uncl. Comamonadaceae*	1%	0%	1%	0%	8%	0%	0%	0%	1%
Proteobacteria	*uncl. Enterobacteriaceae*	0%	0%	0%	0%	0%	0%	0%	66%	8%
Others	*Others*	4%	54%	43%	30%	7%	11%	0%	3%	22%

S6 Table. Effect of mastitis on individual ASVs. Effect size expressed as log2(fold-change) of size-factor-normalized ASV counts. Column "Log Odds Differential Abundance" (log(ODA)) gives the log-odds that the abundance of the ASV differs according to the considered condition. ASVs with log(ODA) ≥ 1 and Taxa with a mean frequency > 1% across samples are shown.

Comparison	ASV	log2(Fold-Change)	Average log2(Abundance)	P-value	Adjusted P-value	Log (ODA)	Feature ID	Genus
Mastitis vs. Healthy	2705	-5.6	0.45	1.5e-39	1.1e-36	68.5	620a844c91124cae407e0f681519c9c7	*Streptococcus*
Mastitis vs. Healthy	2731	-6.5	1.1	6.0e-32	1.3e-30	55.5	f311aaf8e156b56ecee41399a81e2dad	*Streptococcus*
Mastitis vs. Healthy	2690	-3.8	0.49	1.6e-15	6.7e-15	25.5	30e235064f92ef7d67cefe268528fa57	*Streptococcus*
Mastitis vs. Healthy	2686	-7.4	0.56	7.9e-15	3.1e-14	23.9	167f2fc45134213f0950cf5382823fc7	*Streptococcus*
Mastitis vs. Healthy	2689	-6.1	0.45	2.1e-13	7.3e-13	20.6	a5cb2070d12a7e3cc74c1fade629f1db	*Streptococcus*
Mastitis vs. Healthy	2699	-3.2	0.39	1.3e-9	3.7e-9	11.5	ad0285232e3dadd42256c44be5571fa2	*Streptococcus*
Mastitis vs. Healthy	2947	8.9	1	2.6e-26	2.8e-25	50	3e4794d00a0d6be2b067847a554ec70d	*Staphylococcus*
Mastitis vs. Healthy	2918	6.5	0.63	5.9e-17	2.8e-16	28.9	9ecaa8b392f658ea1bf6da401697a504	*Staphylococcus*
Mastitis vs. Healthy	2914	-5.5	0.50	1.2e-16	5.4e-16	28.2	d869795553c6f2ed47d4ac765b71ce63	*Staphylococcus*
Mastitis vs. Healthy	2924	-7.8	0.76	1.3e-9	3.7e-9	12	f515b2bce2c3a00a6e98d57efb16bbf3	*Staphylococcus*
Mastitis vs. Healthy	2897	-3	0.58	8.4e-15	3.2e-14	23.8	062a6836914b958e7d6c97fad350cf1f	*Staphylococcus*
Mastitis vs. Healthy	2894	-3.6	0.58	8.2e-7	0.0000019	5.1	99e66f76afdfe3d9132d97f5ddac9182	*Staphylococcus*
Mastitis vs. Healthy	2905	-5.2	1.2	9.1e-7	0.0000021	5	b125c260eba029c42ac4b63b3ecba5dc	*Staphylococcus*
Mastitis vs. Healthy	2911	5.6	0.42	7.4e-21	4.9e-20	24.7	c94d10830bdd30a9ba1c7122e65d44cd	*Staphylococcus*
Mastitis vs. Healthy	2943	-2.7	0.50	0.000033	0.000068	1.5	142189debc4749557561639aab33b11a	*Staphylococcus*
Mastitis vs. Healthy	1105	-0.56	0.76	6.9e-15	2.7e-14	24.2	92b29c04874b35beca1d1194bcf3439f	*Glutamicibacter*
Mastitis vs. Healthy	1104	-4.4	0.92	2.0e-13	7.0e-13	20.6	2da85155dcafcbdaa7533a7e8af68616	*Glutamicibacter*
Mastitis vs. Healthy	1111	-2.7	0.79	0.000002	0.0000054	4	cdcdc527a76dcf54cddff6c03ee252b9	*Glutamicibacter*
Mastitis vs. Healthy	1594	-4.7	0.58	2.3e-12	7.6e-12	18.1	f794a0e15dfbfd3168fdd64c148dab6c	*Acinetobacter*
Mastitis vs. Healthy	1605	-3.8	0.40	3.2e-31	6.3e-30	56.5	95fb33d2d4b379ea43052fd308b3bbdd	*Acinetobacter*
Mastitis vs. Healthy	1438	-3.6	0.30	2.6e-29	4.3e-28	54.1	282c69a05f11bc96e079bcab4697f7f6	*Pseudomonas*
Mastitis vs. Healthy	1470	-5.6	0.45	3.3e-27	4.2e-26	51.3	75e77495cf3a14881f726cba557d02a7	*Pseudomonas*
Mastitis vs. Healthy	1439	-2.8	0.41	4.0e-28	5.6e-27	51	567a7a2cc2d56b85cc4ef1692e9246eb	*Pseudomonas*
Mastitis vs. Healthy	1472	-3.4	0.47	6.0e-17	2.8e-16	28.9	4973a676cb6f76ec7b6965f91eba57dd	*Pseudomonas*
Mastitis vs. Healthy	1447	-5.8	0.84	1.8e-14	6.8e-14	23.1	c5198ac6c67e0c65a306290ecbdab08a	*Pseudomonas*
Mastitis vs. Healthy	1441	4.7	0.55	3.8e-17	1.8e-16	19.1	bd7a0ad115a02fb7c34defe8414400c5	*Pseudomonas*
Mastitis vs. Healthy	1448	-8.1	1.4	2.1e-10	6.2e-10	13.4	88a8dcfcd5d1265a82ba4a4aa27623b9	*Pseudomonas*
Mastitis vs. Healthy	1476	-6.3	3.6	0.000002	0.0000056	3.6	3c86528743f37651c98350136ce1b8d9	*Pseudomonas*
Mastitis vs. Healthy	1519	-8.5	0.84	8.4e-35	3.2e-33	50	f3698c9106f6a8b82d79912b30f6a9da	*uncl. Enterobacteriaceae*
Mastitis vs. Healthy	1531	-8.7	0.68	5.3e-19	3.0e-18	33.6	ac8527d438803c9e850817444cb84f82	*uncl. Enterobacteriaceae*
Mastitis vs. Healthy	1532	-6.1	0.74	2.2e-18	1.2e-17	32.2	a38c54bf2f4b8d13ca908502be084150	*uncl. Enterobacteriaceae*

Comparison	ASV	log2(Fold-Change)	Average log2(Abundance)	P-value	Adjusted P-value	Log (ODA)	Feature ID	Genus
Mastitis vs. Healthy	825	-7.3	0.88	1.4e-26	1.6e-25	49.7	5cb4a4db4bf5154a9666f20c8ef2ffb4	*Corynebacterium*
Mastitis vs. Healthy	866	-4.7	0.36	1.1e-19	6.3e-19	35.2	950a227b99360cf5ef5393e5b91d3e71	*Corynebacterium*
Mastitis vs. Healthy	862	-3.5	0.44	2.2e-12	7.2e-12	18.3	80481a8c361e5ea5aeaedfdb62bf94a2	*Corynebacterium*
Mastitis vs. Healthy	819	-5.9	1.2	7.2e-10	2.0e-9	12	effc81ffd457fafdbf4fd138c3303703	*Corynebacterium*
Mastitis vs. Healthy	871	-12	1.3	1.0e-9	2.8e-9	11.8	ac15b0dd22b5893f79046070c83d25b8	*Corynebacterium*
Mastitis vs. Healthy	840	3.9	0.73	1.4e-15	6.0e-15	25.7	663f96f8a3e7caa4e7232972fb3457bb	*Corynebacterium*
Mastitis vs. Healthy	813	-2.3	0.38	0.000002	0.0000048	4.5	ebe677d363138d93c5705645ad789d29	*Corynebacterium*
Mastitis vs. Healthy	811	-6	0.43	2.5e-11	7.8e-11	15.7	3897fb4fd655ca611e30aab62814b9eb	*Corynebacterium*
Mastitis vs. Healthy	844	6.2	0.50	4.4e-38	1.1e-35	66.6	979847103a7148a2c1a97d9602e1b221	*Corynebacterium*
Mastitis vs. Healthy	2763	-6.9	2.7	0.00001	0.000021	2.2	27cb8b7e7678914392e0ff83deddbb05	*Lactobacillus*
Mastitis vs. Healthy	2742	-5	0.39	3.6e-21	2.5e-20	38.5	df7aa78d619c4bb8728e3d6365516e56	*Lactobacillus*
Mastitis vs. Healthy	2886	5.3	0.56	1.8e-33	5.2e-32	54.5	00515af64f933ef66984930380ce23f0	*Jeotgalicoccus*
Mastitis vs. Healthy	2888	-6.1	0.77	6.7e-20	4.1e-19	35.7	bdb96f4b6e0e60ca5a7ea89b08ea74da	*Jeotgalicoccus*
Mastitis vs. Healthy	2574	-4.5	0.72	8.0e-16	3.4e-15	26.2	8cc851a2ddefd80afa45eb3354b993f9	*uncl. Sphingomonadaceae*
Mastitis vs. Healthy	2681	12	1.3	5.4e-11	1.6e-10	15	ebda8c5eb205836426a86499f8f84d3e	*Lactococcus*
Mastitis vs. Healthy	1355	-4.6	0.67	3.0e-9	8.0e-9	10.6	f56255163393302af0875ee566abc9e9	*uncl. Comamonadaceae*
Mastitis vs. Healthy	1557	-4.7	0.38	1.8e-20	1.1e-19	37	8624976b800d9183a7938486fd8add96	*Moraxella*
Mastitis vs. Healthy	1563	-4.9	1.1	6.3e-11	1.9e-10	14.4	d6271c7269ef84aa8e5ab5a8c690bf00	*Moraxella*
Mastitis vs. Healthy	1562	-6	1.1	2.6e-10	7.6e-10	13.3	172640323d6f2d482998401ffa20dc26	*Moraxella*
Mastitis vs. Healthy	1549	-6	0.71	1.3e-7	3.1e-7	6.9	a26753b1b850e504b6b507901ce5cbb5	*Moraxella*
Mastitis vs. Healthy	1561	5.7	0.34	1.1e-13	3.8e-13	9.7	a4b9b645a1a601dea25d12d5c2ff01bc	*Moraxella*
Mastitis vs. Healthy	1568	-6.4	2.4	0.000003	0.0000073	3.3	f9b3432811f81de008db7c8e82c829e1	*Moraxella*
Mastitis vs. Healthy	1555	1.4	0.44	6.5e-14	2.4e-13	21.5	7445d44b50503ff580c5747cbd9c61ff	*Moraxella*
Mastitis vs. Healthy	985	-6.6	1.1	1.8e-14	6.9e-14	23	5bfd4c0edb1f3da6e88cb564a44c0573	*Enteractinococcus*
Mastitis vs. Healthy	987	-6.4	0.75	2.0e-22	1.6e-21	41.4	45d8caf1ccb6bc77dc05a8a9b23c769a	*Enteractinococcus*
Mastitis vs. Healthy	993	2.7	0.89	3.9e-11	1.2e-10	15.4	f54d5af5f2d888004753aa4a59b23f62	*Enteractinococcus*
Mastitis vs. Healthy	988	4.3	1.4	0.00002	0.000042	1.7	7d89fb22ae98180210e59e730c2ce809	*Enteractinococcus*
Mastitis vs. Healthy	2572	-4.4	0.93	9.7e-8	2.4e-7	7	7ce648c0b235a7651ea350d732e2f9a9	*Sphingomonas*
Mastitis vs. Healthy	2590	-4.8	1	3.6e-7	8.6e-7	5.6	ddd266e3a80b7011aee0433756e7d915	*Sphingomonas*
Mastitis vs. Healthy	2589	1.5	0.39	0.000001	0.0000023	5	b1c8c0b399a77e081b1750bc1e7964c1	*Sphingomonas*
Mastitis vs. Healthy	2841	-3.8	0.33	1.1e-15	4.6e-15	26	bdeee40a843d1fb6a86d1afb77b0b804	*Atopostipes*
Mastitis vs. Healthy	2823	1.3	0.53	1.8e-20	1.1e-19	36.8	8c67efd486388ebfb54b1e2134af0a6c	*Atopostipes*
Mastitis vs. Healthy	2805	-2.2	0.57	5.1e-15	2.0e-14	24.5	8f8db02b35a3fe28027c5abe5c5c0b53	*Atopostipes*
Mastitis vs. Healthy	2803	2.6	0.74	8.9e-8	2.2e-7	7.3	efbb4a6fc8bd709f5c1744a4135a1b11	*Atopostipes*
Mastitis vs. Healthy	2810	5.8	0.93	2.1e-7	5.1e-7	6.6	334a4a7381950fa44926bd39d45e714d	*Atopostipes*
